# Impact of Dietary Inputs on Carbapenem Resistance Gene Dynamics and Microbial Safety During Bioconversion of Agri-Food Waste and Anaerobic Digestate by *Hermetia illucens* Larvae

**DOI:** 10.3390/genes16080907

**Published:** 2025-07-29

**Authors:** Andrea Marcelli, Alessio Ilari, Vesna Milanović, Ester Foppa Pedretti, Kofi Armah Boakye-Yiadom, Federica Cardinali, Giorgia Rampanti, Andrea Osimani, Cristiana Garofalo, Lucia Aquilanti

**Affiliations:** Dipartimento di Scienze Agrarie, Alimentari ed Ambientali, Università Politecnica delle Marche, Via Brecce Bianche, 60131 Ancona, Italy; a.marcelli@staff.univpm.it (A.M.); a.ilari@staff.univpm.it (A.I.); e.foppa@staff.univpm.it (E.F.P.); kofiboakyeyiadom2@gmail.com (K.A.B.-Y.); f.cardinali@staff.univpm.it (F.C.); g.rampanti@staff.univpm.it (G.R.); a.osimani@staff.univpm.it (A.O.); c.garofalo@staff.univpm.it (C.G.); l.aquilanti@staff.univpm.it (L.A.)

**Keywords:** agri-food waste valorization, *Hermetia illucens*, carbapenem resistance genes, *Enterobacteriaceae*, microbial safety, circular bioeconomy

## Abstract

Background/Objectives: *Hermetia illucens* larvae can efficiently convert agri-food residues into high-protein biomass for animal feed and nutrient-rich frass for soil amendment. However, the potential spread of carbapenem resistance genes (CRGs), which confer resistance to last-resort carbapenem antibiotics, and *Enterobacteriaceae*, common carriers of these genes and opportunistic pathogens, raises important safety concerns. This study aimed to assess the influence of different agri-food-based diets on *Enterobacteriaceae* loads and the CRG occurrence during the bioconversion process. Methods: Four experimental diets were formulated from agri-food residues and anaerobic digestate: Diet 1 (peas and chickpea waste), Diet 2 (peas and wheat waste), Diet 3 (onion and wheat waste), and Diet 4 (wheat waste and digestate). *Enterobacteriaceae* were quantified by viable counts, while five CRGs (*bla_KPC_*, *bla_NDM_*, *bla_OXA-48_*, *bla_VIM_*, and *bla_GES_*) were detected and quantified using quantitative PCRs (qPCRs). Analyses were performed on individual substrates, formulated diets, larvae (before and after bioconversion), and frass. Results: Plant-based diets sustained moderate *Enterobacteriaceae* loads. In contrast, the digestate-based diet led to a significant increase in *Enterobacteriaceae* in both the frass and mature larvae. CRGs were detected only in legume-based diets: *bla_VIM_* and *bla_GES_* were found in both mature larvae and frass, while *bla_OXA-48_* and *bla_KPC_* were found exclusively in either larvae or frass. No CRGs were detected in onion- or digestate-based diets nor in young larvae or diet inputs. Conclusions: The findings suggest that the diet composition may influence the proliferation of *Enterobacteriaceae* and the persistence of CRGs. Careful substrate selection and process monitoring are essential to minimize antimicrobial resistance risks in insect-based bioconversion systems.

## 1. Introduction

The persistent increase in agri-food waste, driven by rapid population growth and urbanization, poses a dual challenge to global food security and environmental sustainability [1,2]. Traditional waste management practices, such as landfilling and incineration, are not only resource exhaustive but also contribute to greenhouse gas emissions and soil degradation. Consequently, the imperative for innovative, circular bioeconomy approaches that transform organic residues into high-value products has never been greater [3]. In this context, insect rearing, particularly of the black soldier fly (*Hermetia illucens*), has emerged as a promising strategy for converting diverse agri-food by-products into valuable biomass, contributing to sustainable protein production and organic waste valorization [4,5]. *H. illucens* larvae exhibit an exceptional capacity to convert heterogeneous organic substrates, ranging from fruit and vegetable wastes to animal manures and anaerobic digestates, into nutrient-rich larval biomass and a residual matrix known as frass. Frass—composed of insect feces, shed exoskeletons (exuviae), and unconsumed substrate—is increasingly recognized as a potent organic soil amendment due to its balanced composition of macro- and micronutrients, beneficial microbial communities, and soil-conditioning properties [6,7]. The application of frass has been shown to improve the soil structure, enhance water retention, and promote plant growth, thereby closing the nutrient loop in agricultural systems [6]. Simultaneously, processed *H. illucens* larvae, rich in high-quality proteins, essential amino acids, and lipids, offer a sustainable alternative to conventional feed ingredients, such as fishmeal and soybean meal, reducing pressure on overexploited marine stocks and deforestation-linked commodity production [8,9,10,11].

Moreover, *H. illucens* larvae can contribute to biogas systems by enhancing the breakdown of organic waste through pre-treatments and by generating residual biomass that can serve as feedstock for anaerobic digestion. The nutrient-rich digestate produced from anaerobic digestion can then be applied to soils as a fertilizer [12], although its high proportion of resistant organic compounds can slow decomposition. Alternatively, this digestate itself can be fed to *H. illucens* larvae, closing the nutrient circle by transforming stubborn organic matter into high-value protein biomass [12,13].

However, despite the environmental and economic benefits of such circular systems, the microbiological safety of the resulting products remains a critical concern, particularly with regard to the potential dissemination of antibiotic resistance (AR) genes [14,15]. Organic residues used as larval feed may harbor resistant microorganisms and genetic determinants of resistance, which could persist through the bioconversion process and ultimately enter the food or environmental chain via insect-derived products. Among AR determinants, carbapenem resistance genes (CRGs) represent a critical threat to public health. Carbapenems constitute last resort β-lactam antibiotics used to treat severe infections caused by multidrug-resistant Gram-negative pathogens. The rapid global dissemination of key carbapenemase-encoding genes, including *bla_KPC_*, *bla_NDM_*, *bla_OXA-48_*, *bla_VIM_*, and *bla_GES_*, has been documented across clinical, agricultural, and environmental settings, undermining therapeutic options and complicating infection control efforts [16]. These enzymes hydrolyze carbapenems and many other β-lactams, conferring high-level resistance and facilitating horizontal gene transfer through mobile genetic elements.

While extensive research has focused on CRGs in hospital effluents and wastewater treatment plants, their potential occurrence, persistence, and fate in insect bioconversion systems remain largely unexplored. Organic residues used as *H. illucens* feed may harbor CRG-carrying bacteria, which could survive or even proliferate during larval digestion, ultimately entering the food chain or the environment via frass and larval biomass. Conversely, recent evidence suggests that *H. illucens* larvae can exert antimicrobial effects through gut mediated competitive exclusion, immune related antimicrobial peptides, and physico-chemical alterations of the substrate that may reduce microbial loads and the AR gene prevalence [4].

Based on these considerations, this study evaluates the microbiological safety of using *H. illucens* larvae to convert various agri-food residues and anaerobic digestate into high-protein feed and nutrient-rich frass suitable as an organic soil amendment. To ensure nutritional suitability, four distinct diets were formulated: peas with chickpea waste (Diet 1), peas with wheat waste (Diet 2), onion with wheat waste (Diet 3), and wheat waste with anaerobic digestate (Diet 4). These substrates were selected and combined to achieve a balanced protein-to-carbohydrate ratio known to support optimal *H. illucens* larval development and biomass gain [17,18].

Changes in bacterial proliferation and the prevalence of CRGs were tracked throughout the bioconversion process. *Enterobacteriaceae* counts were determined using the traditional viable count method, while the abundance of five clinically relevant CRGs (*bla*_KPC_, *bla*_OXA-48_, *bla*_NDM_, *bla*_GES_, and *bla*_VIM_) was quantified via quantitative PCRs (qPCRs). To assess how the diet composition influences the *Enterobacteriaceae* load and CRG persistence, and whether *H. illucens* larvae can mitigate these microbiological risks, analyses were conducted on the individual substrates used for diet formulation, the resulting diet formulations, larvae at early developmental stage prior to diet exposure (young larvae), larvae at the late developmental stage following the bioconversion process (mature larvae), and the resulting frass. This research contributes to the understanding of microbiological safety in insect-based waste recycling systems and provides valuable insights into the potential risk of CRG transmission through the use of *H. illucens*-derived products in agriculture and animal feed.

## 2. Materials and Methods

### 2.1. Experimental Setup and Sampling

Rearing trials of *H. illucens* larvae fed four distinct diets were conducted in a specialized bioconversion facility housed in a modified freight container, equipped with dedicated systems for substrate preparation, larval rearing trays, and environmental control, maintaining a constant temperature of 30 °C and 80% relative humidity.

The agro-industrial residues used for diet formulation, including peas, wheat waste, onions, and digestate, were provided by a consortium of companies from the Marche region (Italy) involved in agricultural production, processing, and the valorization of waste through anaerobic digestion (Covalm, Ortoverde, Covalm Biogas, Cesano di Senigallia (AN), Italy). Only the chickpea substrate was sourced from a regional seed company (PBS Seeds, San Severino Marche, Italy).

To prepare the four different diets, five single substrates were weighed using an industrial platform scale (Model BL30K1, Giorgio Bormac, Carpi (MO), Italy; 30 kg capacity, ±1 g accuracy); ground twice using a double-axle mill, (Model TIGER SHARK 100, Fulltech Instruments S.r.l., Rome (RM), Italy) capable of reducing plant residues and inert materials to an average particle size of approximately 4 mm; and manually mixed. The homogenized formulations (fresh/dry ratios reported in Table 1), 12 kg each, were placed into polypropylene trays (60 × 40 × 23 cm; L × W × H).

Approximately 150,000 young *H. illucens* larvae (4–6 days old) were purchased from SmartBugs s.s. (Villorba, TV, Italy) and evenly distributed into the trays to achieve a larval density of 4.88 larvae/cm^2^. In the experimental setup, certain parameters were standardized, including feed humidity set at 70% and a daily feeding ratio of 100 mg/larva/day. Each diet was tested in 12 replicates, except for the digestate-based diet, which was tested with 6 replicates.

To assess bioconversion efficiency under controlled conditions, the larvae were reared for 10 days. After this period, trays were left for an additional 2 days at 30 °C with humidity control turned off to facilitate subsequent mechanical separation. Samples including individual substrates, diet formulations, young and mature *H. illucens* larvae, and the resulting frass were collected in sterile bags and immediately transported to the laboratory. Although more replicates were initially prepared, they were pooled to generate three biological replicates per sample type for subsequent microbiological and molecular analyses.

### 2.2. Viable Counts

Before analysis, both young larvae (prior to diet exposure) and mature larvae (af-ter the bioconversion process) underwent surface disinfection by immersion in 50 mL of 70% (*v*/*v*) ethanol. This was carried out on a laboratory shaker (VDRL Stirrer with thermostatic cupola, ASAL s.r.l., Milan, Italy) at 150 rpm for 1 min at ambient temperature. Following ethanol treatment, the larvae were rinsed twice in 50 mL of sterile deionized water to eliminate residual ethanol, as described by Milanović et al. [15]. For microbiological analysis, 10 g of each sample, including young and mature larvae, individual diet substrates (peas, chickpea, wheat, onion, and digestate), diet formulations, and frass, was combined with 90 mL of sterile peptone water (1 g/L bacteriological peptone) and homogenized using a Stomacher (400 Circulator, International PBI, Milan, Italy) for 3 min at 260 rpm. The resulting homogenates were subjected to ten-fold serial dilutions and plated on Violet Red Bile Glucose Agar (VRBGA; Merck KGaA, Darmstadt, Germany) for the enumeration of presumptive *Enterobacteriaceae*. Plates were incubated at 37 °C for 24 h. Microbial counts were performed in three biological replicates, each with two technical replicates, and results were expressed as the log of colony-forming units per gram (log CFU/g), reported as mean ± standard deviation.

### 2.3. DNA Extraction and qPCR Quantification of Carbapenem Resistance Genes

Aliquots (1 mL) of the 10^−1^ dilution from each previously prepared homogenate were centrifuged at 14,000 rpm for 10 min using a Rotofix 32A centrifuge (Hettich, Milan, Italy). The resulting pellets were used for total DNA extraction with the E.Z.N.A. Soil DNA Kit (Omega Bio-tek, Norcross, GA, USA), following the manufacturer’s protocol. DNA quantity and purity were assessed using a Nanodrop ND-1000 spectrophotometer (Thermo Fisher Scientific, Wilmington, DE, USA). To verify successful extraction of bacterial DNA, endpoint PCR amplification was carried out using the universal prokaryotic primers 27f and 1495r [19].

qPCR was employed to detect and quantify five CRGs, *bla_KPC_*, *bla_OXA-48_*, *bla_NDM_*, *bla_GES_*, and *bla_VIM_*, according to the protocol described by Milanović et al. [20] and Garofalo et al. [21]. Standard curves for each CRG were created using serial ten-fold dilutions of DNA extracted from five reference bacterial strains [20] each harboring one of the targeted resistance genes. qPCR was conducted on a CFX Connect Real-Time PCR System (Bio-Rad, Hercules, CA, USA), which calculated amplification efficiencies (E) and correlation coefficients (R^2^) from the slope of each standard curve. These standard curves ranged from <1 to 7 log gene copies per reaction, enabling estimation of the detection limits for each gene.

Absolute quantification of CRGs in the samples was achieved by comparing the amplification results with the respective standard curves. Each reaction was set up in a final volume of 10 μL, containing 4 μL of template DNA, 5 μL of Type-it 2X HRM PCR Master Mix (Qiagen, Hilden, Germany), 200 nm of each forward and reverse primer, and nuclease-free water. The thermal cycling conditions were initial denaturation at 95 °C for 5 min, followed by 35 cycles of 95 °C for 20 s, 55 °C for 45 s, and 72 °C for 30 s. Melt curve analysis was performed post-amplification to verify product specificity, with the temperature increasing from 60 °C to 95 °C at a ramp rate of 0.2 °C/s. Results were expressed as log gene copy numbers per gram of sample and reported as mean ± standard deviation from three biological replicates, each analyzed in triplicate (technical replicates).

### 2.4. Statistical Analysis

Statistical differences among sample groups were assessed using one-way analysis of variance (ANOVA), followed by Tukey–Kramer’s Honest Significant Difference (HSD) post hoc test at a significance threshold of *p* < 0.05. All statistical analyses were conducted using JMP software (version 11.0.0, SAS Institute Inc., Cary, NC, USA).

## 3. Results

### 3.1. Viable Counts Results

**Figure 1:**
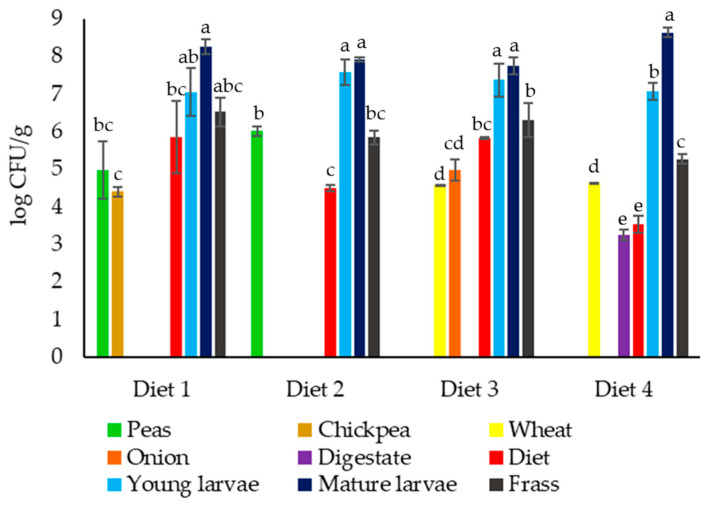
Results of viable counts (log CFU/g) of *Enterobacteriaceae* in samples related to *Hermetia illucens* larvae fed Diet 1 (peas and chickpea waste), Diet 2 (peas and wheat waste), Diet 3 (onion and wheat waste), and Diet 4 (anaerobic digestate and wheat waste) at the beginning and end of the bioconversion process. Values are expressed as means of log CFU/g from three biological and two technical replicates ± standard deviations. For each sample related to the same diet, values with different superscript letters are significantly different (*p* < 0.05) according to the Tukey–Kramer (HSD) test.

Insects harbor a rich and dynamic microbiota, derived from both ingested substrates and resident gut communities, which is essential for their nutrition, development, and health [4,22]. A subset of these microorganisms survives the digestive process, proliferates within the larval gut [23], and is subsequently deposited in frass, thereby linking gut and waste microbiomes [24,25]. Members of the family *Enterobacteriaceae* constitute one of the core taxa within the gut of *H. illucens.* The most frequently detected genera include *Enterobacter*, *Klebsiella*, *Morganella*, *Providencia*, and *Serratia* [25,26]. These bacteria secrete carbohydrases and proteases that facilitate the breakdown of complex polysaccharides and proteins, enhancing larval growth and accelerating the bioconversion of agri-food waste [27,28]. However, several *Enterobacteriaceae* species, particularly within the genera *Klebsiella*, *Escherichia*, *Salmonella*, and *Morganella*, harbor virulence determinants and AR genes that can be transferred to other gut microbes and excreted in frass [29]. Of particular concern is the potential presence of CRGs, which confer resistance to last-resort antibiotics and have been detected in multiple edible insect rearing systems [20]. The dietary substrate composition is known to modulate the abundance of microbial groups, including *Enterobacteriaceae*. High-protein feeds often favor the species from *Enterobacter* and *Morganella* genera, which frequently carry elevated copy numbers of CRGs, whereas fiber-rich diets tend to favor fermentative bacteria with lower resistance loads [30]. Accordingly, in this study, to assess the impact of diet on *Enterobacteriaceae* loads during agri-food waste and anaerobic digestate bioconversion by *H. illucens* larvae, viable counts of *Enterobacteriaceae* were performed in mature larvae fed four distinct diets and in their resulting frass. Moreover, to determine microbial sources and monitor community shifts throughout bioconversion, young larvae prior to diet exposure, each individual substrate used in the diet formulations, and the composite diets were also analyzed. Viable count results are summarized in Figure 1.

For samples related to Diet 1, the lowest *Enterobacteriaceae* count was observed in the chickpea substrate (4.40 log CFU/g), peas (4.97 log CFU/g), and the diet formulation (5.86 log CFU/g), showing no statistically significant differences among them. This aligns with previous research on legume-based substrates, which indicates that *Enterobacteriaceae* levels can range from 4 to 6 log CFU/g, depending on processing methods and storage conditions [31]. The highest load of this microbial group was detected in mature *H. illucens* larvae (8.26 log CFU/g). However, no statistically significant differences were observed when compared to young larvae. Additionally, no statistically significant differences were found between the Diet 1 formulation used for rearing *H. illucens* larvae and the frass obtained at the end of the rearing process.

Regarding Diet 2 (peas and wheat waste), *Enterobacteriaceae* were not detected in the wheat waste substrate, indicating that their presence in Diet 2 originates from the peas. The highest loads of *Enterobacteriaceae* were found in mature (7.91 log CFU/g) and young (7.58 log CFU/g) *H. illucens* larvae. The Diet 2 formulation and the frass obtained after rearing showed lower loads (4.49 and 5.85 log CFU/g, respectively), with no statistically significant differences between them.

In regards to Diet 3 (onion and wheat waste), the highest loads of *Enterobacteriaceae* were found in mature (7.75 log CFU/g) and young (7.37 log CFU/g) *H. illucens* larvae, with no statistically significant differences among them. The substrates used for the formulation of Diet 3 were characterized by the lowest counts, with 4.58 log CFU/g found in the wheat waste, followed by the onion (4.98 log CFU/g). The letter results are in line with what was previously reported by Almou et al. [32], showing *Enterobacteriaceae* loads in onion samples ranging from about 2.0 to 4.0 log CFU/g. Even if the *Enterobacteriaceae* load was higher in frass (6.30 log CFU/g) with respect to the initial Diet 3 formulation (5.82 log CFU/g), no significant differences were observed between them.

Finally, regarding the Diet 4 formulation (wheat waste and digestate), the lowest *Enterobacteriaceae* load was found in the digestate (3.25 log CFU/g). This value aligns with studies on raw digestates collected from biogas plants in France, which reported *Enterobacteriaceae* counts ranging from 1.0 to 4.0 log CFU/g [33]. Conversely, a recent study by Wójcik-Fatla et al. [34] reported higher values, with an average *Enterobacteriaceae* concentration of approximately 6 log CFU/g in digestate samples. The highest load of presumptive *Enterobacteriaceae* was found in mature *H. illucens* larvae (8.64 log CFU/g), showing a statistically significant increase compared to young *H. illucens* larvae (7.07 log CFU/g). Similarly, the frass was characterized by a higher count of *Enterobacteriaceae* compared to the initial Diet 4 formulation.

Overall, in our study, young *H. illucens* larvae were characterized by *Enterobacteriaceae* loads between 7.05 and 7.58 log CFU/g. In mature larvae(after bioconversion process), counts varied by diet, ranging from 7.75 log CFU/g for those reared on Diet 3, composed of onion and wheat waste, to 8.64 log CFU/g for those fed a digestate and wheat waste mixture (Diet 4). These findings are consistent with Brulé et al. [4], who reported *Enterobacteriaceae* loads ranging from 2.9 to 9.7 log CFU/g in *H. illucens* larvae grown on a variety of substrates, ranging from traditional diets (cereals, fruits, vegetables) and vegetable agri-food by-products to different foodstuffs and meat, with the highest counts associated with nutrient-rich, microbiologically complex substrates such as digestate and meat residues. Conversely, Osimani et al. [35] observed lower *Enterobacteriaceae* levels in larvae reared on substrates that included coffee silverskin or varying amounts of *Schyzochitrium limacinum* or *Isochrysis galbana*.

Although there was a general increase in *Enterobacteriaceae* levels in mature larvae fed Diets 1–3, these differences were not statistically significant and likely reflect a natural developmental variation rather than a strong microbial shift. In contrast to plant-based diets, Diet 4 induced a statistically significant increase in *Enterobacteriaceae* in mature larvae, suggesting diet-driven microbial proliferation rather than a developmental effect. These findings are likely to reflect the specific microbial contamination intrinsic to digestate, a substrate known to contain *Enterobacteriaceae* strains well adapted to anaerobic conditions [4,36]. While *H. illucens* larvae are generally effective at suppressing these bacteria in plant-based diets (Diets 1 to 3) through competitive exclusion and the production of antimicrobial peptides [37], our data suggests that digestate-derived strains can overwhelm these natural defense mechanisms. This observation supports the findings of Brulé et al. [4], who noted that substrates such as vegetable by-products and digestate can introduce microbial loads that exceed the larvae’s capacity for control.

Regarding the frass, *Enterobacteriaceae* loads ranged from 5.27 log CFU/g in those corresponding to Diet 4 to 6.53 log CFU/g in those associated with Diet 1. These values align with the findings of Brulé et al. [4] and Wynants et al. [25], who reported *Enterobacteriaceae* levels between 1.0 and 9.7 log CFU/g in frass derived from rearing *H. illucens* larvae on various substrates. Osimani et al. [35] observed slightly lower counts in frass, ranging from 3.7 to 5.0 log CFU/g. Variations in rearing conditions, including differences in sampling times during the rearing cycle, feeding rates, and larval densities, can vary between studies and may influence the observed microbiological counts in the frass [25,38]. Similarly to the trends observed in larvae, although *Enterobacteriaceae* counts were consistently higher in the frass than in the initial diet formulations used for rearing, no statistically significant differences were found between the initial diet formulations 1 to 3 and their corresponding frass. In contrast, Diet 4 resulted in an increase in *Enterobacteriaceae* counts in the resulting frass. For Diets 1 to 3, which are composed of plant-based residues such as peas, chickpea waste, wheat waste, and onion waste, the absence of significant changes in *Enterobacteriaceae* levels between the initial substrates and the frass suggests that the larvae effectively modulate microbial proliferation without introducing additional contamination. This observation is consistent with Brulé et al. [4], who noted that larvae reared on low-risk substrates such as cereals, fruits, or vegetables maintain a microbial equilibrium, even though a complete pathogen elimination is not achieved. If the frass is to be utilized as a plant fertilizer or soil amendment, a heat treatment becomes essential to lower *Enterobacteriaceae* levels. According to European Union (EU) Regulation (EU) No. 2021/1925 [39], insect frass must undergo thermal processing at 70 °C for 60 min. This procedure has been shown to effectively reduce *Enterobacteriaceae*, including *Escherichia coli*, to levels below 1.0 log CFU/g, as documented in previous studies [40].

### 3.2. qPCR Quantification of Carbapenem Resistance Genes

Tracking AR genes in *H. illucens* larvae used for animal feed and in the resulting frass applied as soil amendment is essential to mitigate the potential dissemination of resistance within the food chain and the environment. AR genes present in larvae may be transmitted to livestock and eventually to humans, whereas frass can act as a conduit for resistance genes in the soil, with possible repercussions on plant health and surrounding ecosystems. Special attention is given to CRGs, as carbapenems are the last-resort antibiotics in human medicine, and the spread of these genes poses a serious threat to public health by limiting treatment options for multidrug-resistant infections. This issue is recognized in European legislation, including Commission Regulation (EU) 2017/893 [41], on the use of insects in feed, and Regulation (EU) 2019/1009 [42], on fertilizer products, as well as in the European Food Safety Authority (EFSA’s) guidance [43], which supports a One Health strategy to address AR. In this context, we examined the impact of different dietary inputs on the CRG composition in both larvae and frass and assessed whether the larvae could contribute to reducing microbial hazards.

The qPCR standard curves created for each of the five CRGs under study exhibited good amplification efficiencies, ranging from 90% to 110%, with R^2^ values greater than 0.99 for all reactions. The detection limit was below one log gene copy per reaction for all the tested genes.

Across all tested diets, CRGs were detected infrequently and inconsistently among the samples (Table 2).

No CRGs were identified in the individual feed ingredients, the diet formulations, the young larvae analyzed prior to diet exposure, or in any of the samples associated with Diets 3 and 4. For Diet 1, *bla*_VIM_ was detected in both the frass and mature larvae, at 8.05 and 6.67 log gene copies/g, respectively, with significantly higher levels observed in the frass. Mature larvae also harbored *bla_OXA-48_* and *bla_GES_*, quantified at 5.44 and 7.65 log gene copies/g, respectively. In the case of Diet 2, frass samples contained *bla_KPC_* (8.17 log gene copies/g) and *bla_GES_* (8.66 log gene copies/g). The latter was also quantified in mature larvae (8.14 log gene copies/g), along with *bla_OXA-48_* (5.43 log gene copies/g). *bla_GES_* was the only gene detected in both frass and mature larvae, with a statistically higher abundance in the frass.

The use of carbapenems is forbidden in food-producing animals worldwide [44]. Nonetheless, increasing reports of carbapenem-resistant bacteria in food-producing animals, aquaculture, seafood, companion animals, and wildlife indicate the growing dissemination of carbapenem resistance across diverse ecosystems [16]. To the best of our knowledge, no scientific publications have yet addressed the occurrence of CRGs in *H. illucens* larvae. Consequently, direct comparisons or broader contextual discussions remain limited. The only relevant study to date investigating CRGs in edible insects [19] analyzed ready-to-eat grasshoppers and mealworms originating from Europe (Belgium and the Netherlands) and Asia (Thailand). Consistent with our findings, they reported only a sporadic detection of the same CRGs investigated in our study. While the *bla_GES_* gene emerged as the most frequently detected gene in our samples, being present in both the frass and larvae related to Diet 2 and in larvae fed Diet 1, Milanović et al. [20] did not detect it in any of the analyzed insect samples. Furthermore, in our study, *bla_OXA-48_* was found in mature larvae fed Diets 1 and 2 but not in frass, while Milanović et al. [20] reported *bla_OXA-48_* at a low frequency (3%) in mealworms but at a significantly higher frequency (57%) in grasshoppers. *bla_VIM_* was detected in our samples exclusively in frass and mature larvae reared on Diet 1 (peas and chickpeas), whereas Milanović et al. [20] reported this gene in only 2 out of 30 grasshopper samples (7% positivity) and none of the mealworm samples, suggesting that VIM-type carbapenemases are rare in insect matrices and may be selected under specific diet-related microbial conditions. While Milanović et al. [20] found no KPC-type genes in either grasshoppers or mealworms, we detected *bla_KPC_* exclusively in the frass resulting from the conversion of Diet 2. Finally, *bla_NDM_*, which Milanović et al. [20] quantified in 10% of mealworms and 27% of grasshopper samples, was not detected in any of our larval or frass samples. These findings suggest that the presence and distribution of CRGs in insect matrices are highly variable, and the detection of specific CRGs associated with particular diets highlights the possible influence of dietary compositions on the acquisition and accumulation of AR genes in *H. illucens* larvae and their rearing environment.

Notably, in the present study, no CRGs were detected in young larvae prior to diet exposure or in any of the diet formulations used for rearing. Their subsequent detection in mature larvae and frass after the bioconversion process suggests that CRG-carrying bacteria may have been present in the system at levels below the qPCR detection threshold and became enriched during larval development. This enrichment could have resulted from selective pressures or microbial interactions within the larval gut that favored the proliferation or persistence of initially low-abundance CRG-harboring bacteria. The exclusive detection of CRGs in samples associated with legume-based diets (peas with chickpeas or wheat) further implies that specific substrate compositions may promote the proliferation or retention of these resistant bacteria. Conversely, the absence of CRGs in diets containing onion or anaerobic digestate indicates that these substrates may not support the growth or survival of such microorganisms. However, this interpretation becomes more complex when considering that the digestate-based diet (Diet 4) led to a significant increase in the total load of presumptive *Enterobacteriaceae* in both the mature larvae and frass. This indicates that while the digestate may not support the presence of CRG-carrying bacteria specifically, it still promotes the growth of *Enterobacteriaceae* in general. Therefore, digestate appears to promote the proliferation of non-CRG-harboring members of this family, highlighting the need to distinguish between the overall bacterial growth and the presence of specific antimicrobial resistance determinants when evaluating feed safety and microbiota dynamics.

Previous findings showed that the microbial composition of feed substrates can significantly influence both the gut microbiota of *H. illucens* larvae and the associated resistome [27]. Similarly, Milanović et al. [20] hypothesized that CRGs detected in edible insects are more likely the result of external contamination via feed substrates, handling surfaces, equipment, or post-harvest processing, rather than antibiotic use during rearing. They further noted that species-specific feeding behaviors (e.g., grasshoppers consuming foliage versus mealworms consuming cereal-based substrates) and associated husbandry practices may account for the distinct patterns of the CRG occurrence observed among different insect species.

From an environmental perspective, the high CRG loads detected in frass, up to 8.66 log gene copies/g in Diet 2, raise concerns about the potential enrichment of soil and crops with CRGs following its use as fertilizer. Studies on manure-based amendments have shown that AR genes can persist in soils, colonize plant phyllospheres, and potentially enter the food chain [45]. To limit the spread of AR genes, including CRGs, EU Regulation 2021/1925 [39] mandates the thermal processing of frass before its agricultural application. While it may not completely degrade free DNA, the goal of the thermal treatment is to eliminate viable microbial vectors capable of harboring resistance genes.

## 4. Conclusions

The present study suggests that the dietary substrate composition influences both the abundance of *Enterobacteriaceae* and the occurrence of CRGs in *H. illucens* larvae and their frass. Plant-based diets (peas, chickpeas, wheat, onion) supported moderate *Enterobacteriaceae* loads (4–8 log CFU/g) that did not change significantly through larval development or in the resulting frass. In contrast, a digestate-based diet (Diet 4) produced significantly higher *Enterobacteriaceae* counts in mature larvae and frass, suggesting that microbial proliferation was driven by the diet rather than by larval development. Digestate-associated bacteria may persist despite the larvae’s intrinsic capacity to modulate gut microbial loads.

The qPCR quantification of CRGs revealed that all detected genes were confined to legume-based diets: *bla_VIM_* and *bla_GES_* were present in both larvae and frass obtained after the bioconversion process, while *bla_OXA-48_* and *bla_KPC_* were restricted to either frass or larvae. No CRGs were detected in young larvae analyzed prior to diet exposure, in the feed substrates alone, or in the onion- and digestate-based diets. These findings suggest that the presence of CRGs in mature *H. illucens* larvae is likely associated with certain feed compositions that may promote the proliferation or retention of CRG-harboring bacteria. From an applied perspective, the detection of high CRG loads—up to 8.66 log gene copies/g, in frass and up of 8.14 log gene copies/g in mature larvae intended for soil amendment and feed, respectively—raises environmental and public health concerns.

Our findings highlight the importance of diet selection in insect rearing, not only to optimize growth and the nutritional value but also to mitigate the risk of disseminating AR genes. Future research should prioritize the identification of specific microbial reservoirs of CRGs in feed substrates, the elucidation of CRG acquisition mechanisms within the larval gut, and the optimization of both substrate pre-treatments and rearing conditions to reduce the spread of antimicrobial resistance along insect-based feed and fertilizer value chains.

## Figures and Tables

**Table 1:** Composition of the diets used for *H. illucens* larvae bioconversion trials.

Diet	Substrates (Fresh + Dry)	Ratio (Fresh/Dry)
1	peas + chickpea	3.5:1
2	peas + wheat	3.5:1
3	onion + wheat	3:1
4	liquid digestate + wheat	2:1

**Table 2:** Log copy number of carbapenem resistance genes per gram of samples related to *Hermetia illucens* larvae fed four different diets.

Diet	Sample	Carbapenem Resistance Genes (log Gene Copies/g ± Standard Deviation)				
		*bla_KPC_*	*bla_OXA-48_*	*bla_NDM_*	*bla_GES_*	*bla_VIM_*
1	Peas	n.d.	n.d.	n.d.	n.d.	n.d.
	Chickpea	n.d.	n.d.	n.d.	n.d.	n.d.
	Diet (peas + chickpea)	n.d.	n.d.	n.d.	n.d.	n.d.
	Young *H. illucens* larvae	n.d.	n.d.	n.d.	n.d.	n.d.
	Frass	n.d.	n.d.	n.d.	n.d.	8.05 ± 0.02 ^a^
	Mature *H. illucens* larvae	n.d.	5.44 ± 0.02	n.d.	7.65 ± 0.01	6.67 ± 0.02 ^b^
2	Peas	n.d.	n.d.	n.d.	n.d.	n.d.
	Wheat	n.d.	n.d.	n.d.	n.d.	n.d.
	Diet 2 (peas + wheat)	n.d.	n.d.	n.d.	n.d.	n.d.
	Young *H. illucens* larvae	n.d.	n.d.	n.d.	n.d.	n.d.
	Frass	8.17 ± 0.02	n.d.	n.d.	8.66 ± 0.01 ^a^	n.d.
	Mature *H. illucens* larvae	n.d.	5.43 ± 0.02	n.d.	8.14 ± 0.06 ^b^	n.d.
3	Onion	n.d.	n.d.	n.d.	n.d.	n.d.
	Wheat	n.d.	n.d.	n.d.	n.d.	n.d.
	Diet 3 (onion + wheat)	n.d.	n.d.	n.d.	n.d.	n.d.
	Young *H. illucens* larvae	n.d.	n.d.	n.d.	n.d.	n.d.
	Frass	n.d.	n.d.	n.d.	n.d.	n.d.
	Mature *H. illucens* larvae	n.d.	n.d.	n.d.	n.d.	n.d.
4	Digestate	n.d.	n.d.	n.d.	n.d.	n.d.
	Wheat	n.d.	n.d.	n.d.	n.d.	n.d.
	Diet 4 (digestate + wheat)	n.d.	n.d.	n.d.	n.d.	n.d.
	Young *H. illucens* larvae	n.d.	n.d.	n.d.	n.d.	n.d.
	Frass	n.d.	n.d.	n.d.	n.d.	n.d.
	Mature *H. illucens* larvae	n.d.	n.d.	n.d.	n.d.	n.d.

n.d., not detected. For each parameter, values with different superscript letters are significantly different (*p* < 0.05) according to the Tukey–Kramer’s (HSD) test.

## Data Availability

The original contributions presented in this study are included in the article. Further inquiries can be directed to the corresponding author.

## Abbreviations

The following abbreviations are used in this manuscript:

AR	Antibiotic Resistance
CRGs	Carbapenem Resistance Genes
qPCR	Quantitative Polymerase Chain Reaction
EU	European Union
EFSA	European Food Safety Authority
